# Simultaneous Accessory Pathway and AV Node Mechanical Block

**DOI:** 10.1016/s0972-6292(16)30672-6

**Published:** 2013-09-01

**Authors:** Daniel Garofalo, Alfonso Gomez Gallanti, David Filgueiras Rama, Rafael Peinado Peinado

**Affiliations:** Unidad de Electrofisiología y Arritmias, Hospital Universitario "La Paz", Madrid, Spain

**Keywords:** Orthodromic reciprocating tachycardia, Accessory pathway, Cryoablation, Atrioventricular node, Block

## Abstract

We report a clinical case of a 22-year-old female referred to our institution due to palpitations and preexcitation. Her ECG suggested a right superior paraseptal accessory pathway (AP), which was localised during the electrophysiological study at the superior paraseptal region in close proximity to the His recordings. Reproducible orthodromic reciprocating tachycardia was induced by atrial pacing with extrastimuli. Cryo-mapping performed in the area of earliest atrial activation was not able to terminate the tachycardia. A second attempt, slightly more posterior, caused mechanical block of the AP, which rendered the tachycardia non-inducible. More pressure with the ablation catheter determined a Wenckebach type supra-hisian AV block, which was transient but reproducible. Given this finding no ablation was done. Simultaneous block to the AP and the atrioventricular node has rarely been reported using radiofrequency energy. However, to our knowledge this phenomenon has not been previously reported in large series using cryo-thermal energy.

A 22-year-old female, with no relevant medical history apart from being a smoker, was referred to our Unit due to episodes of palpitations and the finding of pre-excitation on her ECG. This had been noted 4 years prior during a visit to the Emergency Department due to palpitations and dizziness. As the episode was self-limited no arrhythmia was documented at that point. From then on she referred to a history of palpitations, with episodes that on average occurred monthly and never exceeded a minute in duration. The palpitations had a sudden onset and offset, had no particular trigger and were not accompanied by other symptoms. No tachycardia was ever recorded on her ECG, and she never took any antiarrhythmic drugs. Her ECG (Figure 1) suggested a right superior paraseptal accessory pathway (AP). Her echocardiogram was normal, and a Holter study showed persistent pre-excitation but did not reveal any episodes of tachycardia. An exercise stress test showed sinus rhythm throughout and disappearance of the delta wave at heart rates above 170 beats per minute.

Given her age and symptomatic status, an electrophysiology study was performed for potential ablation of the AP. Venous access was obtained via the right femoral vein. A decapolar catheter was placed in the coronary sinus, while two quadripolar catheters were used for recording in the His region and the right ventricular apex. At the beginning of the study the patient showed intermittent pre-excitation, mostly 2:1. However, while placing the His catheter the delta wave disappeared. Under these circumstances, the HV interval measured 40 milliseconds (ms). Ventriculo-atrial (VA) conduction was present and was not decremental. Atrial activation was concentric, with earliest activity recorded at the His catheter. The minimum 1:1 VA conduction was at 300 ms, and the retrograde effective refractory period was 250 ms for the AP and 240 ms for the AV node, both at a cycle length of 400 ms. Antegrade conduction over the AP could not be assessed due to the mechanical block caused by the His catheter. Minimum 1:1 atrio-ventricular (AV) conduction occurred via the AV node at 250 ms. There was no evidence of dual AV node physiology. An atrial train followed by an extra-stimulus coupled at 260 ms reproducibly induced a regular tachycardia with narrow QRS complexes and an average cycle length of 260 ms. During tachycardia, the HV interval was 51 ms, and earliest atrial activation was recorded in the superior paraseptal region (Figure 2), a similar pattern to that observed during ventricular pacing. In that area, continuous electrical activity was observed. The VA interval was 69 ms, and entrainment from the right ventricular apex was not possible: either the tachycardia was not sustained or degenerated into atrial fibrillation (AF). With a diagnosis of atrio-ventricular reciprocating tachycardia (AVRT) due to a right superior paraseptal AP, ablation was undertaken.

Given the proximity of the circuit to the normal conduction system, the decision was made to perform cryo-mapping before attempting ablation, using a 6-mm tip Freezor Xtra catheter (Medtronic, Inc.). Recordings with the ablation catheter during AVRT showed a His potential of less than 0.1 mV at the site of earliest atrial activation. Cryo-mapping in that area was not able to terminate the tachycardia. With atrial pacing during AVRT, AF was induced twice. The first episode required electric cardioversion; the second episode was not sustained. Ventricular pre-excitation was not observed in either episode of AF. A second attempt at cryo-mapping was performed slightly posterior to the initial site, where no His potential was recorded. By this time antegrade conduction over the AP had resumed intermittently, with the odd QRS complex showing pre-excitation. However, mechanical block occurred again, which determined antegrade and retrograde block of the pathway. The latter was suggested by non-inducibility of the tachycardia despite pacing and isoproterenol infusion (while originally the induction was extremely reproducible) and by VA conduction showing decremental properties. At the same site, more pressure with the ablation catheter determined a Wenckebach type AV block (Figure 3). A His catheter recording confirmed this block to be supra-hisian (Figure 4); it resolved after a few minutes but was reproducible. Given this finding and the AP properties, no ablation was done. The patient was discharged on no anti-arrhythmic drugs, and after one year her symptoms have remained stable and have had little impact on her quality of life.

## Discussion

Catheter-based cryo-thermal ablation became available in the late 1990s, following its successful use in the setting of arrhythmia surgery.[1] During cryo-mapping, cooling of the AV node results in reversible AV block, practically eliminating permanent AV block as a complication of ablation of arrhythmias close to the normal conduction system.[2] The stability gained by the adherence of the catheter tip to the endocardium also helps predict the effectiveness and safety of the lesion.[3]

Mechanical block of conducting tissue has been described in different substrates. It has been recognised that right-sided and septal AP are more prone to trauma by the catheter, presumably due to their subendocardial nature.[4,5] With regards to the AV node, young age (<35 years) has been found to be an independent predictor of mechanical trauma caused by the catheter.[6] Simultaneous block to the AP and the AV node has rarely been reported. In the study by Belhassen et al [7] using radiofrequency, two patients with right anteroseptal AP experienced trauma to both the pathway and the AV node. In a large series using cryo-thermal energy,[8] this phenomenon has not been reported. It is worth noting that the cryo-energy catheter is generally more rigid than most conventional radiofrequency catheters. This rare case of simultaneous AP and AV node mechanical block during cryo-mapping in a superior paraseptal pathway highlights the difficulty that mechanical block and proximity to the normal conduction system can cause during an ablation.

## Figures and Tables

**Figure 1:**
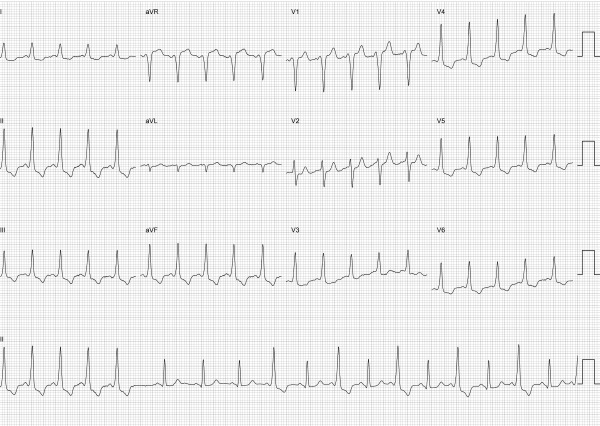
Baseline 12-lead ECG at the beginning of the electrophysiology study showing intermittent pre-excitation. A positive/negative QRS in V1 and R>S in lead III suggested a right superior paraseptal location.

**Figure 2:**
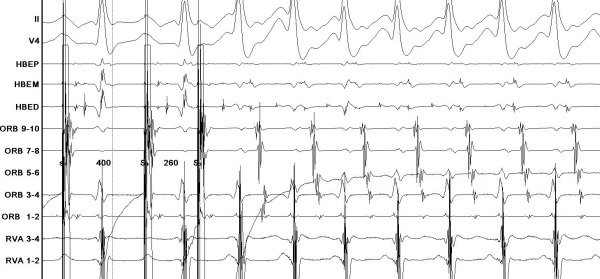
Induction of atrio-ventricular reciprocating tachycardia with a single extrastimulus from the atrium. Earliest atrial activation can be seen at the proximal pair of electrodes at the His catheter, which shows continuous activity and near absence of His electrogram. HBEP, HBEM, HBED: His catheter from proximal to distal. ORB: decapolar catheter placed in the coronary sinus. RVA: right ventricular apex catheter.

**Figure 3:**
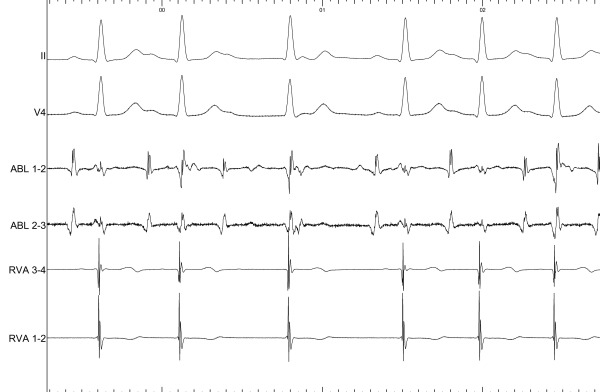
Wenckebach-type AV block caused by pressure with the ablation catheter, showed at a sweep speed of 67 mm/sec. ABL: ablation catheter. RVA: right ventricular apex catheter.

**Figure 4:**
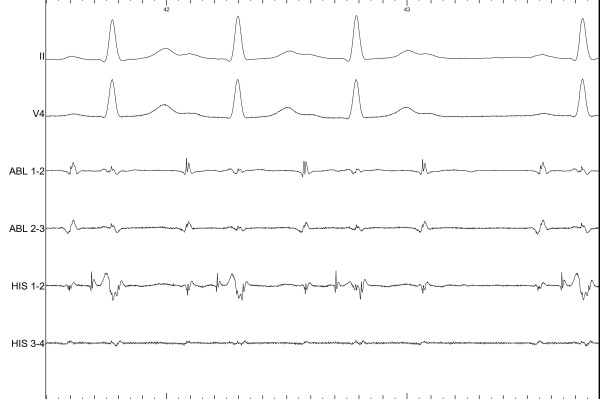
A His catheter recording confirms a supra-hisian block. Note the absence of a His potential following the atrial activity after the third QRS complex.

## References

[R1] Skanes A (2004). Cryoablation: Potentials and Pitfalls. J Cardiovasc Electrophysiol.

[R2] De Sisti A (2012). Cryoablation of Atrioventricular Nodal Reentrant Tachycardia: A Clinical Review. PACE.

[R3] Gaita F (2003). Safety and Efficacy of Cryoablation of Accessory Pathways Adjacent to the Normal Conduction System. J Cardiovasc Electrophysiol.

[R4] Novick T (1978). Catheter-Induced Block in Accessory Pathways. Circulation.

[R5] Chiang C (1994). Incidence, Significance, and Pharmacological Responses of Catheter-Induced Mechanical Trauma in Patients Receiving Radiofrequency Ablation for Supraventricular Tachycardia. Circulation.

[R6] Topilski I (2007). Catheter-Induced Mechanical Trauma to Fast and Slow Pathways during Radiofrequency Ablation of Atrioventricular Nodal Reentry Tachycardia: Incidence, Predictors, and Clinical Implications. PACE.

[R7] Belhassen B (1999). Catheter-Induced Mechanical Trauma to Accessory Pathways during Radiofrequency Ablation: Incidence, Predictors and Clinical implications. J Am Coll Cardiol.

[R8] Bastani H (2010). Cryoablation of superoparaseptal and septal accessory pathways: a single centre experience. Europace.

